# Three-Dimensional Printing of Poly(3-hydroxybutyrate-co-3-hydroxyvalerate) [P(3HB-co-3HV)] Biodegradable Scaffolds: Properties, In Vitro and In Vivo Evaluation

**DOI:** 10.3390/ijms241612969

**Published:** 2023-08-19

**Authors:** Ekaterina I. Shishatskaya, Aleksey V. Demidenko, Aleksey G. Sukovatyi, Alexey E. Dudaev, Aleksey V. Mylnikov, Konstantin A. Kisterskij, Tatiana G. Volova

**Affiliations:** 1 Institute of Biophysics SB RAS, Federal Research Center “Krasnoyarsk Science Center SB RAS”, Akademgorodok, 50/50, 660036 Krasnoyarsk, Russia; shishatskaya@inbox.ru (E.I.S.); kraysolnca@mail.ru (A.V.D.); a.sukovatiy@yandex.ru (A.G.S.); alex15-96@mail.ru (A.E.D.); 2 School of Fundamental Biology and Biotechnology, Siberian Federal University, Svobodnyi Av. 79, 660041 Krasnoyarsk, Russia; kisterski@yandex.ru; 3 Clinical Hospital “RZD-Medicine”, Lomonosov Street, 47, 660058 Krasnoyarsk, Russia

**Keywords:** 3D printing, FDM technology, P(3HB-co-3HV) copolymer, filaments, 3D scaffolds, structure, mechanical strength, biocompatibility in vitro, osteoplastic properties in vivo

## Abstract

The results of constructing 3D scaffolds from degradable poly(3-hydrosbutyrpate-co-3-hydroxyvalerate) using FDM technology and studying the structure, mechanical properties, biocompatibility in vitro, and osteoplastic properties in vivo are presented. In the process of obtaining granules, filaments, and scaffolds from the initial polymer material, a slight change in the crystallization and glass transition temperature and a noticeable decrease in molecular weight (by 40%) were registered. During the compression test, depending on the direction of load application (parallel or perpendicular to the layers of the scaffold), the 3D scaffolds had a Young’s modulus of 207.52 ± 19.12 and 241.34 ± 7.62 MPa and compressive stress tensile strength of 19.45 ± 2.10 and 22.43 ± 1.89 MPa, respectively. SEM, fluorescent staining with DAPI, and calorimetric MTT tests showed the high biological compatibility of scaffolds and active colonization by NIH 3T3 fibroblasts, which retained their metabolic activity for a long time (up to 10 days). The osteoplastic properties of the 3D scaffolds were studied in the segmental osteotomy test on a model defect in the diaphyseal zone of the femur in domestic Landrace pigs. X-ray and histological analysis confirmed the formation of fully mature bone tissue and complete restoration of the defect in 150 days of observation. The results allow us to conclude that the constructed resorbable 3D scaffolds are promising for bone grafting.

## 1. Introduction

Three-dimensional printing (3DP), also known as additive manufacturing (AM), is a current technology for the manufacturing of three-dimensional physical objects, layer by layer, from a digital model, directly from a computer-aided design (CAD) file. Today, the use of additive manufacturing has moved far beyond prototyping and represents a universal method with high potential in transforming and improving many traditional industries [1,2,3]. Such 3D printing technology is widely used in many fields (construction, electronics, biomedicine, aerospace, automotives [4], fabrics [5], and food [6]). According to different implementation principles, 3D printing technology is divided into several categories [7,8,9], including fused filament fabrication (FFF), direct ink writing (DIW), stereolithography appearance (SLA), selective laser sintering (SLS), laminated object manufacturing (LOM), and liquid deposition molding (LDM). To implement these technologies, various materials are used, the characteristics of which must correspond to the chosen 3D printing technology. The authors of [10] divided the materials used for 3D printing into organic, metallic, inorganic non-metallic, and composite materials. Today, in connection with the prospects for the development of “green” and low-carbon energy and the need for the efficient use of renewable natural resources for 3D printing technology, natural materials of various origins are actively considered [9]. The authors found that the number of publications and references on 3D printing technology using natural materials from 2015 to 15 May 2023 had increased by an order of magnitude and it had become one of the key areas for the development of additive technologies.

The prospects and relevance of additive technologies are illustrated by the growing number of publications. A bibliometric analysis of the Scopus database performed by the authors of [3] revealed 30,700 bibliographic articles published between 2000 and 2019 and 157,132 patents filed for 3DP/AM.

The development of additive technologies is associated with the potential to solve the key problems of modern healthcare, aimed at individual patient treatment. Today, 3D printing, which potentially makes it possible to recreate not only tissue fragments but also individual parts of organs and entire organs, is considered the latest technology in reconstructive medicine, including the construction of scaffolds for cell and tissue engineering and implants for the reconstruction of damaged tissues and organs. Three 3D printing technologies have received particular development: laser printing (SLA), powder technologies (3DP), and melt extrusion through a nozzle (FDM). Stereolithography (SLA) is one of the most well-known methods that has become commercial. The first system, called “three-dimensional printing techniques”, was developed in the early 1990s at the Massachusetts Institute of Technology (Cambridge, MA, USA) by Sachs et al. [11]. The layering process allows the fabrication of complex anatomical structures, but the process itself is quite expensive to use and only some polymers compatible with UV curing are suitable for it [12]. An alternative method is the use of a selective laser sintering (SLS) system, in which the binder jet is replaced by a laser beam that melts selected samples of powdered material [13]. The layer thickness and powder particle size are the main factors affecting the final surface structure of the matrices [14]. FDM systems constitute the third family of additive technologies [15]. The technology consists of the fusion of pre-prepared thermoplastic polymer filaments (or powder) and their subsequent melting with the application of a molten polymer material according to a given scheme [16]. FDM systems are the most popular rapid prototyping technologies that are used to manufacture complex matrices of various geometries [16]. The development of FDM technology largely depends on the availability of materials with adequate thermal properties and thermal stability. Therefore, the range of materials studied and used in this technology is limited. These are well-known polymers of lactic acid [17], copolymers of lactide with glycolide [18], as well as polycaprolactone [19,20] and acrylonitrile butadiene styrene (ABS) [21].

It should be noted that the success of the development of 3D printing technologies in general is largely associated with the search for and attraction of new materials with the necessary properties that ensure the creation of highly functional implants and structures with a full range of necessary characteristics. An important position among degradable polymers is occupied by polymers of microbiological origin, called polyhydroxyalkanoates (PHAs). PHAs are a family of biodegradable thermoplastic polymers with different chemical structures and different physicochemical properties [22,23,24,25,26,27]. PHAs have high potential as biocompatible and resorbable materials for the manufacture of various biomedical products. These are suture materials, nonwoven disposable products, controlled drug delivery systems, scaffolds for tissue engineering, implants for reconstructive medicine, and vascular stents [28,29,30,31,32,33]. Successful examples of the use of PHA products as dermal equivalents for the restoration of skin and soft tissue defects, osteoplastic materials, for the restoration of the pericardium, blood vessels, heart valves, etc., have been described [34,35,36,37,38,39,40,41].

A PHA is a relatively “young” biomedical material, the active research of which was launched in the late 1980s and early 1990s, much later than that of polylactides. At the same time, already in 2007, the most elastic and low-crystalline member of this family (poly(4-hydroxybutyrate)) received FDA approval for clinical use as a suture material [42], and later as meshes and film systems for uroplasty [43]. Despite the undoubted promise of polyhydroxyalkanoates, their use for 3D printing has been poorly developed. This is due to the fact that the most studied and available representative of PHAs, polymer-3-hydroxybutyric acid (poly-3-hydroxybutyrate (P[3HB])), is characterized by high crystallinity and does not crystallize with the formation of an ordered structure. This complicates its processing, and the resulting products do not have elasticity and become brittle with time [44,45,46]. P(3HB), as with many other types of PHAs, during crystallization from melts, forms rather large spherulites [47] and at low rates [48] does not contribute to the production of high-quality products. This therefore limits the use of these polymers, especially for high-temperature processes, including FDM technology.

In this regard, the results obtained by Professor A. Kovalcik et al. [49] are important. They studied the viscosity, thermal properties, and thermal stability of several commercial types of PHAs and showed that P(3HB) and P(3HB-co-3HV) degraded noticeably during the re-treatment of the melt, in contrast to P(3HB-co-3HHx), which has greater thermal stability for use in FDM. The authors summarized the possibility of expanding the range of biodegradable polymeric materials for 3D printing by attracting various representatives of the PHA family. Professor Ipsite Roy noted in his fundamental review [33] that, in recent years, commercial companies have begun developing filaments from blends of PHAs with known synthetic polymers, mainly polylactides [50], but there are still very few publications on the production of filaments for 3D printing from pure PHAs. In the work of Wu et al., the preparation of filaments or composite materials based on PHAs in a mixture with polylactides and/or wax was studied, including wear resistance, biodegradability, and weather resistance depending on the composition and ratio of components [51,52]. Regarding 3D printing technologies using pure PHAs, isolated works are known. These are SLS for the printing of PHBV and Ca-P/PHBV bone tissue scaffolds [53] and lattice constructions from 3HB-co-3HHx to repair a defect in the rabbit radius [54]. It should also be noted that the representative review of D. Puppi and F. Chiellini and their research made a great contribution to the development of additive technologies using 3D printing, SLS and FDM, and various polymer materials, including PHAs of various compositions [55].

The justified interest in PHAs has led to the development and appearance on the market of commercial products that are blended filaments of PHAs and polylactides for 3D printing. These are the commercial products ColorFabb (PLA/PHA) and 3D PrintLife (PLAyPHAbTM) [56,57]. However, the types and amounts of PHAs used in these filaments are unknown [50]. It should be noted the great contribution of Professor Ipsita Roy and the scientific team of the Department of Materials Science and Engineering, Faculty of Engineering, University of Sheffield (UK), to the development of work in the field of medical materials science on polyhydroxyalkanoates and, in particular, the assessment of the prospects for the use of PHA in 3D printing processes [33,58,59]. These groundbreaking works explored commercial ColorFabb “Natural” PHA/PLLA filaments and designed and 3D-printed scaffolds for a variety of biomedical applications. It has been shown that these pioneer scaffolds (3D prototypes) are suitable for restoring various tissues and elements of internal organs; they demonstrated high biocompatibility in the culture of eukaryotic cells of various origins, as well as the absence of noticeable cytotoxicity and inflammatory and immune reactions in experiments in vivo. Summarizing the results obtained, the authors emphasized the high potential of commercial PLA/PHA filaments for FDM printing and also showed that PHAs used in blended filaments are not inferior to polylactide in a number of characteristics. This indicates the undoubted prospects for the use of various types of PHAs in additive technologies. In recent years, there has been an increasing number of publications confirming this [60,61,62,63,64,65].

This paper presents the results of the three-dimensional printing of poly(3-hydroxybutyrate-co-3-hydroxyvalerate) (P(3HB-co-3HV)) biodegradable scaffolds and studying their properties, including mechanical characteristics, biocompatibility assessment in vitro, and osteoplastic properties in vivo.

## 2. Results and Discussion

Using the P(3HB-co-3HV) copolymer, the processes of manufacturing filaments, 3D scaffolds, and their characteristics were studied. The process included the preliminary production of filaments by extrusion and the subsequent printing of 3D scaffolds using FDM technology. The complete technological chain included a series of successive and interrelated stages: (1) the isolation of polymer material samples from a bacterial biomass; (2) obtaining a granulate from the melt; (3) the extrusion production of filaments from the melt; (4) FDM printing and scaffolding. At these stages, the polymer material was subjected to heating, melting, extrusion, and stretching. Therefore, it was necessary to answer the question of how the basic properties of the polymer material changed in this multi-stage process.

### 2.1. Changing the Properties of P(3HB-co-3HV) in the Process of Obtaining Filaments and 3D Printing

Table 1 shows the properties of the original copolymer and the products of processing from it by stage. The results confirm the current idea that PHAs undergo thermal degradation during heat treatment. This is clearly illustrated by the indicators of the average molecular weight, which, compared with the initial values of Mw for the copolymer (530 kDa), decreased step by step to 390 kDa for the scaffolds, i.e., by 40%. At the same time, the polydispersity index practically did not change.

The temperature characteristics of the polymeric material underwent changes to a lesser extent. The thermal degradation temperature (T_degr_) and melting point (T_melt_) remained virtually unchanged. The differences lay in the formation during the first heating and melting (in the process of obtaining a granulate) of several peaks in the melting region. At the same time, the areas of appearance of peaks were practically comparable for all samples obtained by stage: the first peak in the region 147–161; the second (main peak) in 168–172, and the third in the region of 183–185 °C. The enthalpy of melting (H, J/g) decreased in the process of obtaining the granulate from 98.3 to 65.2 J/g, but became higher (73.1 and 80.3 J/g) for the filament and scaffold, respectively. Upon cooling, all samples had two crystallization peaks at all stages, including those of the original copolymer. For the initial copolymer and filament, these peaks had a small size near the zero line on the thermograms. It should be noted that the crystallization shifted to lower temperatures during the thermal processing of P(3HB-co-3HV). The initial copolymer crystallized at 67 °C, and the filament and 3D scaffold at 59 and 54 °C, respectively. A decrease in the glass transition temperature was also noted, from 1.1 °C for the initial copolymer and granulate to 0.3 °C for the filament after the second heating–melting and to 0.2 °C after the third heating–melting for the finished scaffolds.

The resulting slight changes in the thermal behavior of the polymer material during processing and a significant drop in molecular weight, however, made it possible to obtain the desired products—filaments for 3D printing and scaffolds.

### 2.2. Characteristics of P(3HB-co-3HV) Filaments

**Figure 1:**
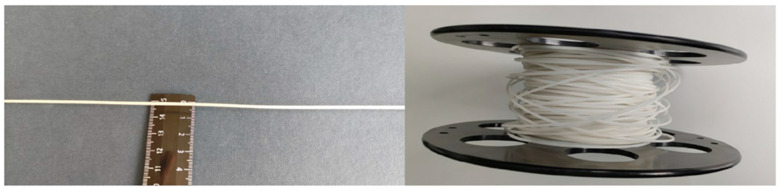
Photo of the obtained filaments.

The filament obtained by extrusion of the P(3HB-co-3HV) melt was a monolithic single-core rod with a diameter of 1.7 ± 0.12 mm (Figure 1). The developed technique made it possible to obtain uniform-in-thickness and defect-free samples of filaments that were hundreds of meters long.

The indices of mechanical strength during the tensile test of P(3HB-co-3HV) filaments were the Young’s modulus, tensile strength, and elongation at break, which were 765.33 ± 123.91; 23.37 ± 0.93 MPa; and 4.83 ± 0.24%, respectively. When tested in bending, the Young’s modulus was 4219.66 ± 172.182 MPa, the ultimate strength was 64.90 ± 2.95 MPa, and the flexural strain was 3.71 ± 0.5%. Considering the mechanical bending tests of filaments, it is necessary to emphasize the features of this type of test. The strength measured for a material in bending is not equivalent to the strength measured in one-dimensional tension. During the manufacture of samples (especially by extrusion), a certain orientation of the polymer C-chain occurs. However, if a load is applied in the direction of the predominant chain orientation, this can result in higher stress values and lower strain values.

One of the first examples of obtaining PHA filaments for 3D printing is described in [66]. A Metabolix PHA sample (polymer composition not specified) was used for the extrusion production of filaments using EX2 Filabot technology at 170 °C in comparison with polylactide and ABS; a significant effect of the melt temperature on the PHA extrusion process was established and filaments with a diameter of 2.85 ± 0.1 mm were obtained. In [67], the manufacture of filaments from a mixture of two different PHA samples from Metabolix, P(3HB) and P(3HB-co-4HB) copolymer, was studied using a Filabot Wee filament extruder. By varying the temperature parameters and extrusion speed, more than 12 linear feet of filament with a diameter of 1.3 mm and 2.9 mm were produced; the longest single thread was 4 feet 5 inches. Data on the mechanical properties of the threads were not presented in these works.

An analysis of more recent publications has shown significant variation in the performance of filaments made from PHAs of various chemical compositions using various 3D printing technologies. Filaments made from commercial copolymer P(3HB-co-3HHx) from Kaneka (Westerlo-Oevel Belgium) had a Young’s modulus of 260 ± 28.0, tension strength of 22.1 ± 4.0, and an elongation at break of 22 ± 4.0%. This was inferior to the values for polylactide filaments, respectively, 670 ± 17.1 and 46.2 ± 1.6 MPa and 24 ± 2.6% [49]. PLA/PHA filaments from ColorFabb had a diameter of 3.5 mm, Young’s modulus of 180.7 ± 27 MPa, elongation at break of 30.4 ± 2.1%, and tensile strength of 56.7 ± 0.9 MPa [59]. The Young’s modulus was found to be approximately six times lower than that of filaments from a similar blended material (lactide/PHA) in the work of Gonzalez et al. [56]; however, the elongation at break was approximately 10 times higher. The authors attributed this to differences in the printing technology and process temperature. Thus, the P(3HB-co-3HV) filaments for 3D printing obtained in this work correspond to the published data.

### 2.3. Characteristics of 3D Scaffolds

**Figure 2:**
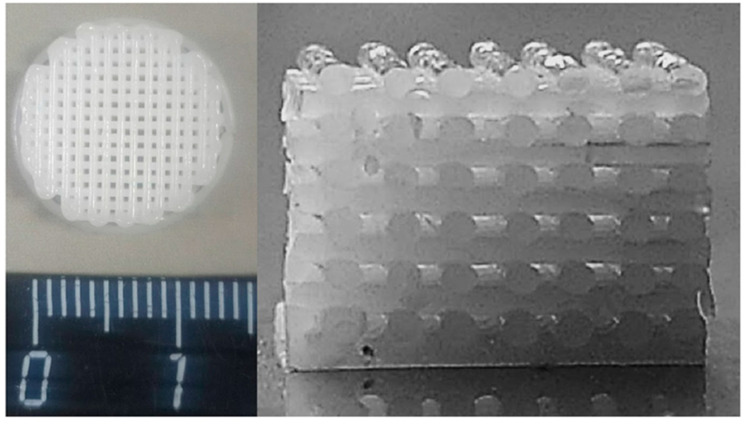
Photo of a 3D scaffold from P(3HB-co-3HV): general view (**left**) and longitudinal section (**right**).

In this study, 3D scaffolds of a cellular cylindrical shape with a diameter of 13 mm and a height of 4 mm were obtained (Figure 2). According to the IUPAC classification, scaffolds are rigid, homogeneous, macroporous solids. The scaffold structure consisted of 12 perpendicular successive layers formed by cylindrical filaments with a diameter of 417.27 ± 32.45 µm, located at a distance of 396.25 ± 54.63 µm. The porosity of the scaffolds was 65 ± 2%.

The porosity and interconnectedness of the pores in scaffolds are important characteristics, since they play an important role in cell adhesion, proliferation, and vascularization [38,39]. It is believed that the ideal architecture of a cell scaffold for bone tissue engineering should have pores with a diameter of 150–500 μm and an interconnected porosity of 60–80% [40]. In terms of porosity, the obtained 3D scaffolds met these requirements.

**Figure 3:**
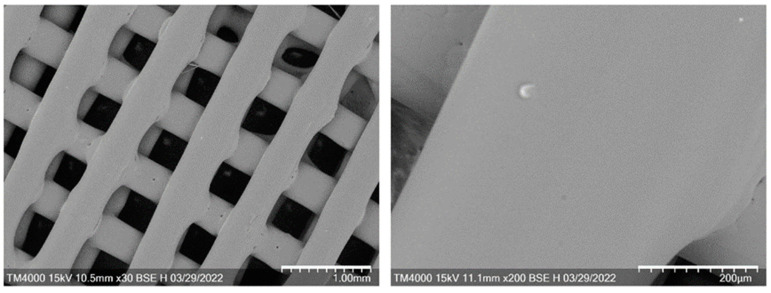
SEM images of 3D P(3HB-co-3HV) scaffold: bar 1 mm (**left**) and 200 µm (**right**).

SEM images of the scaffolds (Figure 3) showed traces of thermal fusion at the intersections of perpendicular layers, which ensured the integrity of the scaffold structure. The surface was smooth, even, and without pronounced relief.

The mechanical characteristics of implants intended for bone grafting depend not only on the type of bone for which they are intended and its location in the body, but also on the orientation of the applied load. Therefore, measurements of compressive strength are important in physical–mechanical testing. In this work, the parameters of the mechanical strength of 3D scaffolds were measured under compressive loads in mutually perpendicular directions (perpendicular to the layers of the scaffold), as well as parallel to them.

The 3D scaffolds obtained by the FDM technology from P(3HB-co-3HV) during a compression test had the following mechanical strength indicators: when a load was applied perpendicular to the scaffold layers, the Young’s modulus was 207.52 ± 19.12 MPa, and the compressive stress tensile strength was 19.45 ± 2.10 MPa. When the load was applied parallel to the layers, the indicators were somewhat different and amounted to 241.34 ± 7.62 MPa and 22.43 ± 1.89 MPa, respectively. In [68], mechanical compression tests were performed on porous model implants made of calcified material, in which the Young’s modulus was in the range of 0.01–3 GPa, and the compressive strength (tensile strength) was from 0.1 to 30 MPa. The authors of this work compared the mechanical strength indicators of the developed implants with the strength of human bone tissue of various structures and functions and concluded that they were suitable for the regeneration of bone defects. Thus, the values of the mechanical characteristics of 3D scaffolds made of P(3HB-co-3HV) obtained in this work, in terms of compressive strength, indicate the suitability of their use for bone grafting of the cortical bone layer, which can be considered as a transversely isotropic material. This conclusion also corresponds to the requirements for implants for bone grafting formulated in [53,69].

The first PHA scaffolds were produced by SLS printing technology [70]. Since powder was used in this process, the pore size in the resulting scaffolds depended on the size of the powder as well as the degree of fusion of the powder particles. The influence of the PHA type on the mechanical properties of 3D scaffolds has been described in several works. In [60], 3D scaffolds obtained from the P(3HB) homopolymer had compressive strength and Young’s modulus values of 0.72 ± 0.04 and 4.91 ± 0.30 MPa, respectively. This is inferior to the performance of 3D scaffolds obtained in the present work from the P(3HB-co-3HV) copolymer and exceeds the data of [53], which describe scaffolds obtained by the SLS technology also from the P(3HB-co-3HV copolymer), but filled with calcium phosphate. Higher mechanical strength was recorded for 3D scaffolds obtained by FDM technology from the P(3HB-co-3HHx) copolymer, which is characterized by higher thermal stability compared to P(3HB) and P(3HB-co-3HV) copolymers [49]. The authors of this work also described a change in the mechanical strength indices of 3D scaffolds made by FDM technology from polylactide and P(3HB-co-3HHx) copolymer, compared to the filaments from which these products were obtained. The elastic modulus of the PLA and P(3HB-co-3HHx) frameworks was higher than that of the original filaments by 125 and 269%, respectively. The opposite effect was recorded for the tensile strength and elongation at break, which significantly decreased in 3D scaffolds when processing filaments in the 3D printing process.

The developed 3D scaffolds obtained in this work also had slightly reduced mechanical strength characteristics compared to the filaments from which they were obtained by FDM technology, while, in terms of mechanical compressive strength, they corresponded to the results obtained in the works of colleagues [49,53] and are promising for use as implants for the regeneration of human bone tissue defects [69].

### 2.4. In Vitro Assays of 3D Scaffolds

**Figure 4:**
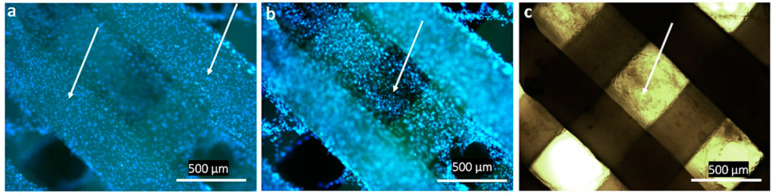
Fluorescence (**a**,**b**) and light microscopy (**c**) of proliferating NIH 3T3 fibroblasts (arrows) on the surface (**a**) and in cells (**b**,**c**) of 3D scaffolds.

The biological compatibility of the 3D scaffolds was studied in vitro in an NIH 3T3 mouse fibroblast culture. The cytocompatibility and ability of the scaffolds to support cell proliferation on the surface and in the cells between the filaments was evaluated. Light and fluorescence microscopy images of cultured cells on 3D scaffolds showed that the cells formed a monolayer over the entire surface (photo 1 in Figure 4) and migrated and populated the spaces between the filaments (photos 2–3 in Figure 4).

**Figure 5:**
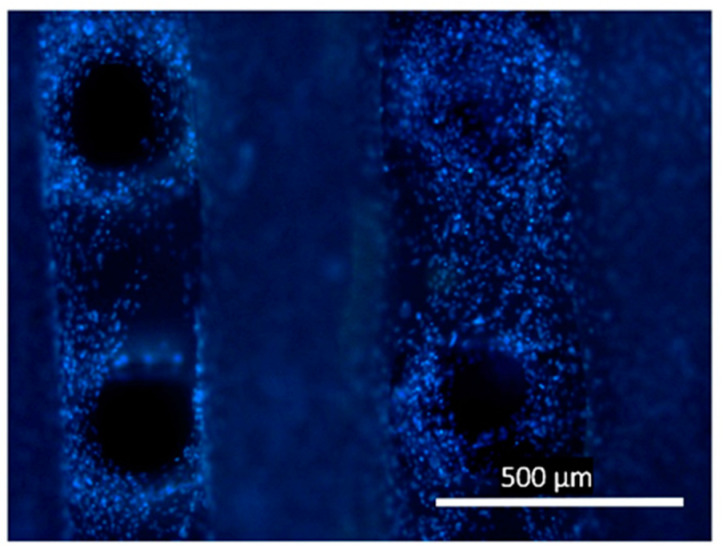
Fluorescent imaging (DAPI stain) of NIH 3T3 fibroblast growth on 3D scaffolds and in cells.

The results of the DAPI staining analysis (Figure 5) also showed cell growth on the surface of the 3D scaffold and in the cells between the scaffold filaments. In this case, there was a gradual radial closure of the entire space between the filaments.

**Figure 6:**
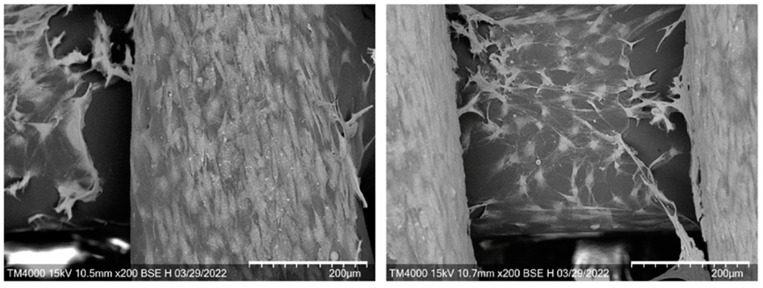
SEM images of NIH 3T3 fibroblasts cultured on the surface of 3D scaffolds on the 3rd day. Bar 200 µm.

SEM images (Figure 6) showed the presence of a large number of spindle-shaped elongated fibroblasts, completely covering the surface of the scaffold. The cells spread over the surface until confluence and migrated to the underlying layers, gradually covering the surfaces of the filaments and filling the space between them.

The viability of fibroblasts was assessed in the MTT test. The results showed that the metabolic activity of 3T3 fibroblasts cultured on 3D scaffolds was maintained for a long time. This is evidence of the high biocompatibility and suitability of the resulting P(3HB-co-34HV) scaffolds for the growth of eukaryotic cells. The number of metabolically active cells gradually increased and on the 10th day of cultivation was 21.37 × 10^5^ cells/cm^2^.

An important aspect of the fabrication of 3D scaffolds is the provision of conditions for increased adhesive properties of the surface with respect to eukaryotic cells and subsequent proliferation. Thus, work [71] describes the positive effect of filling 3D frameworks with hydroxyapatite (HA) particles. Fully biodegradable and nanocomposite 3D scaffolds for bone tissue regeneration, consisting of poly(ε-caprolactone) (PCL) reinforced with hydroxyapatite nanoparticles, significantly improved the compressive mechanical properties, and they also had a fully interconnected pore system, which contributed to the increase in the biological properties of scaffolds. Active colonization of the scaffolds by human mesenchymal stem cells was observed on both types of scaffolds made from PCL and PCL/HA. However, cell adhesion and spreading was better on mixed PCL/HA scaffolds compared to PCL polymer scaffolds. Work [72] describes another technique to positively influence cell attachment and development on 3D polycaprolactone scaffolds. The authors used a unique approach for the 3D assembly of cell constructs by constructing magnetic scaffolds using advanced magnetic materials, mixing bioresorbable Fe-doped hydroxyapatite (FeHA) particles into poly(ε-caprolactone). The 3D scaffolds designed according to the original author’s rules, which were characterized by short (100–200 µm), strong magnetic gradients, were able to orient and trap magnetized cells on the selected side of the scaffold fibers. As a result of targeted action, two populations of cells (mesenchymal stem cells (MSC) and human umbilical vein endothelial cells (HUVEC)) were separated on opposite sides of the fibers of the magnetic osteogenic scaffold. This solution was aimed at providing reconstruction of bone microarchitectonics with adequate properties, including vascularization of the developing bone tissue.

Positive examples of testing scaffolds obtained by 3D printing from PHAs in cell culture in vitro are described in a number of works. Thus, scaffolds obtained by the FDM technology from the P(3HB-co-3HHx) copolymer in the culture of mouse embryonic fibroblast cells (ATCC CRL-1658 NIH/3T3, Manassas, Virginia, USA) for 96 h showed no cytotoxicity and high proliferation potential [49]. The number and morphology of cells were comparable in two types of 3D scaffolds—the original uncoated and coated with gelatin—while exceeding those on the polylactide scaffold. A similar result was obtained in [73] in the study of P(3HB-co-3HHx) and PLA matrices in an L929 fibroblast culture. The 3D scaffolds from P(3HB) were studied in the primary culture of rat long bone mesenchymal cells in vitro (rat bone marrow stem cells—RBMSCs) [59,74]. Cell viability and proliferation were comparable on all types of scaffolds, regardless of the presence or absence in the matrix of osteogenic growth peptide (OGP). An indirect assessment of calcium precipitation at different periods (from 3 to 21 days) did not reveal statistically significant differences.

### 2.5. In Vivo Assays of 3D Scaffolds

The choice of PHA type is due to the fact that short-chain PHAs (SCL-PHA-P3HB homopolymer and P(3HB-co-3HV) copolymers) are considered the most suitable for bone regeneration, due to their high crystallinity, Young’s modulus, and tensile strength. It is also known that filling scaffolds made of P(3HB) and P(3HB-co-3HV) with fillers increases the mechanical properties of the products made from them and approaches the properties of bone [59,74]. Therefore, 3D P(3HB-co-3HV) scaffolds were obtained in the present study and studied in a segmental osteotomy model in an animal experiment.

Both animals satisfactorily underwent surgery and emerged from anesthesia. The restoration of appetite in animals and the motor activity of the operated limbs occurred on days 3–5. Swelling and hyperemia in the area of operation and implantation of the scaffold completely stopped on days 7–9. During the entire observation period, the animals normally gained weight and were active.

**Figure 7:**
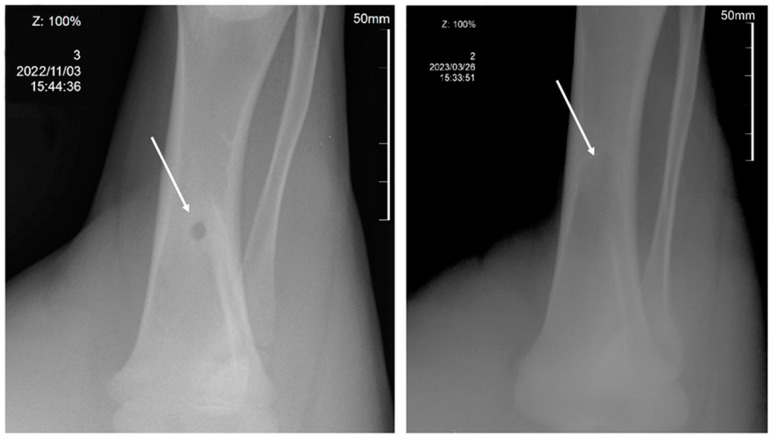
Data from X-ray studies of restoration of a model bone tissue defect in experimental animals using 3D implants from P(3HB-co-3HV): 1—3 days after surgery (**left**); 2—after 150 days (**right**).

X-ray studies performed 150 days after the operation and implantation of 3D scaffolds showed that the model defect was completely closed (Figure 7). In both animals, the restoration of the anatomical structure of the bone was confirmed radiographically.

After the observation was completed, the animals were withdrawn from the experiment. Euthanasia was performed by the intravenous administration of a lethal dose of 150 mg/kg ketamine HCl (Panpharma, Luitré-Dompierre, France).

**Figure 8:**
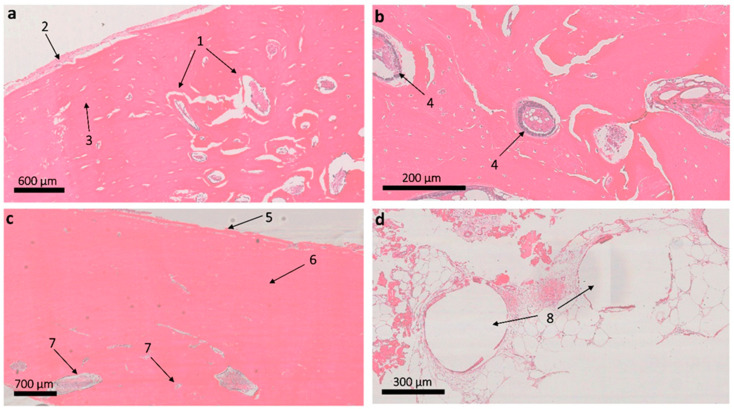
The femur in the area of implantation of the 3D scaffold made of P(3HB-co-3HB): (**a**): 1—newly formed bone with secondary osteons; 2—periosteum; 3—a layer of unchanged external girdle bone plates; (**b**): 4—active osteoblasts in secondary osteons; (**c**): 5—periosteum; 6—layer of external girdle bone plates; 7—osteon layer; (**d**): the implant standing area in the soft tissue around the bone, 8—voids from the crumbled remains of the polymer material formed during the histological preparation of sections, corresponding to the cross-sections of the residual filaments at the implant location. Hematoxylin–eosin staining.

According to histological studies, the regeneration of the model bone tissue defect could be considered consistent. In histological preparations (Figure 8), at the site of a model defect in the femoral tissue at the site of implantation of the 3D scaffold, a moderate decrease in the thickness of the layer of external girdle bone plates is observed due to the ingrowth of a large number of secondary osteons from the osteon layer into it. In the deeper sections, the secondary osteons are smaller and more closely spaced. In some areas in the zone of implantation in osteoblasts in secondary osteons, they are enlarged in size, with large nuclei. Secondary osteons are formed in the zone of defects in the compact substance of the bone to replenish the structural integrity of the bone, as well as in the course of life during the functional restructuring of bone tissue. The site of the defect in the area of implantation of the material is replaced by mature bone lamellar tissue of the usual histological structure without osteomyelitis.

**Figure 9:**
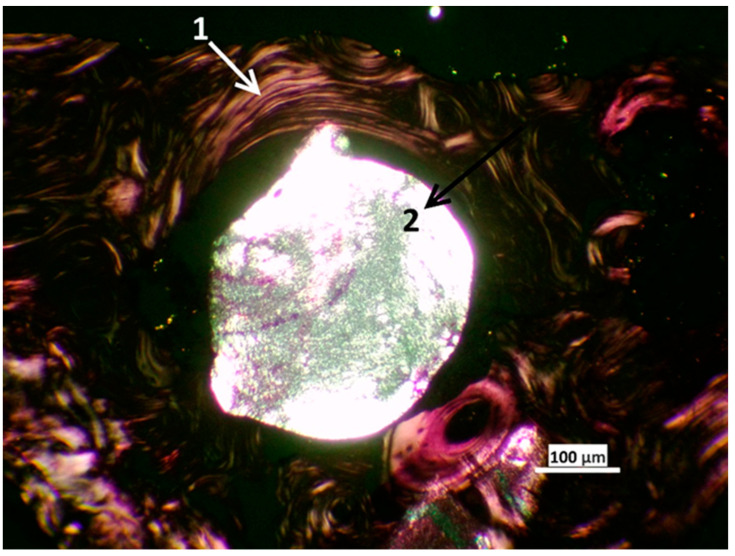
Remains of the polymeric material of the implant in the area of implantation under polarizing microscopy: 1—bone plates of the surrounding bone tissue, 2—an implant in the bone tissue. Bar 100 µm.

In the area of the closed defect, preserved particles of polymeric material were found in the newly formed bone tissue in the form of single, optically transparent, rounded fragments with well-pronounced anisotropic properties during polarizing microscopy. These inclusions were detected on the inner side of the compact bone substance at the transition to the cancellous bone substance. The polymeric material of the residual fragments of the 3D scaffold (diameter in cross-section 313.98 + 16.41, which is 25% lower than the diameter of the cross-section of the original filament in 3D printing) was surrounded by lamellar bone tissue without the formation of a connective tissue capsule and morphological signs of inflammation (Figure 9). In the soft tissue adjacent to the bone, single fragments of the polymer material were also found in the form of thin strips with pronounced anisotropic properties during polarizing microscopy. No inflammatory reaction around the remnants of the material was recorded.

An analysis of publications shows that, recently, scaffolds obtained by 3D printing using PHA have begun to be investigated as bone-replacing implants. An example of a P(3HB) bone replacement scaffold that was printed using selective laser sintering (SLS) was described [70]. To improve the biocompatibility of such P(3HB) scaffolds, Saska et al. covered the surface with osteogenic growth factors by simple physical absorption [60]. In another work, Duan et al. improved the osteoconductive properties of 3D P(3HB-co-HV) scaffolds by increasing calcium phosphate [53]. The 3D printing of bone-replacing implants has been studied using a medium-chain copolymer, P(3HB-co-3HHx), and BG [75]. The 3D scaffolds were tested in vivo in rats and shown to stimulate bone regeneration 8 weeks after implantation. Yang et al. [76] fabricated, studied, and positively evaluated composite scaffolds by 3D printing from a P(3HB-co-3HHx) melt.

The results of the experiment and the initial evaluation of the osteoplastic properties of 3D scaffolds made of P(3HB-co-3HV) allow us to conclude that it is promising for reconstructive osteogenesis. The developed and studied 3D implants, which did not contain any drugs and osteogenesis stimulants, nevertheless ensured the formation of full and mature bone tissue and the complete restoration of the defect. 

## 3. Materials and Methods

### 3.1. Scaffold Material

A copolymer formed by monomers of 3-hydroxybutyrate and 3-hydroxyvalerate (P(3HB-co-3HV)), including monomers 3HB 15 mol.%, was used. The polymer was synthesized at the Institute of Biophysics of the Siberian Branch of the Russian Academy of Sciences by the natural strain *Cupriavidus eutrophus* B-10646 [77], according to the authors’ technology [78]. Bacteria were cultivated in a Schlegel salt medium [79] using glucose (20 g/L) as a carbon substrate in a periodic two-stage regime, with limitation with nitrogen (NH_4_Cl) at the first stage and in a nitrogen-free medium at the second stage. For the synthesis of 3HV monomers, potassium valerate (Sigma-Aldrich, Saint Louis, MO, USA) was used as a precursor.

### 3.2. PHA Recovery from Cell Biomass

The polymer was extracted from the cell biomass with dichloromethane; the resulting extract was concentrated on an R/210V rotary evaporator (Büchi, Flawil, Switzerland) and then precipitated with ethanol. Repeating the procedures of polymer dissolution and reprecipitation ensured the removal of impurities, obtaining homogeneous samples. The chemical purity of the samples was detected using a 7890A gas chromatograph equipped with a 5975C chromatograph–mass spectrometer (Agilent Technologies, Santa Clara, CA, USA). The polymer was dried in a fume hood at room temperature for 72 h.

### 3.3. PHA Chemical Composition

The purity of the PHA and chemical contents was determined by chromatography of the methyl esters of fatty acids after the methanolysis of purified polymer samples using a 7890A chromatograph–mass spectrometer (Agilent Technologies, Santa Clara, CA, USA) equipped with a 5975C mass detector (Agilent Technologies, Santa Clara, CA, USA) [80].

### 3.4. Obtaining Filaments for 3D Printing

A flaky dry sample of P(3HB-co-3HV) copolymer isolated from cells was used to prepare a granulate (Fimar, Rimini, Italy). The granules were loaded into a Brabender E 19/25 D (Brabender, Duisburg, Germany) single-screw extruder with four heating zones. A spinneret with a diameter of 1.5 mm was used to form the filament. The temperature in the extruder by heating zones was in the range of 159–170 °C; the rotation speed of the extruder screw was 15 min^−1^. The filament was stretched and oriented using a Brabender belt conveyor (Brabender, Duisburg, Germany).

### 3.5. Design and Fabrication of 3D Scaffolds

For 3D modeling, a software package for CAD/CAM modeling, Inventor 2022 (Autodesk, USA), was used. Samples were printed using the layer-by-layer deposition of material (FDM) technology using a Hercules 2018 3D printer (Imprinta, Krasnoyarsk, Russia) with a modified extruder with a vortex blowing system for extrusion masses [81], which helps to reduce thermal deformation during 3D printing and the thermal zoning of the print area. The creation of the motion vector of the extrusion head and the slicing and preparation of the machine code (g-code) for the 3D printer were carried out using the Slic3r open-source program.

The 3D scaffolds were printed using FDM technology, layer by layer, at a right angle, drawing a pattern consisting of parallel lines, which were applied in subsequent layers with a 90° rotation relative to each previous layer. The layer height was 0.25 mm; the diameter of the extrusion nozzle was 0.5 mm. The speed of movement of the extrusion head (hot end) during filling with a layer was 60 mm/sec, the extrusion temperature was 175–180 °C, and the temperature of the heated substrate (bed) was 90–110 °C.

### 3.6. Morphology and Physicochemical Properties of Filaments and 3D Scaffolds

The morphology of the obtained products was recorded visually and using electron microscopy using a Hitachi TM4000 scanning electron microscope (Hitachi, Minato-ku, Tokyo, Japan). The filament width and spacing were recorded using the Digital Image Analysis Software Package (a free and open-source software package for scientific analysis, editing, and bitmap processing) named Image J v1.53k. The physicochemical properties of the original copolymer sample, at the stages of preparation and of the obtained filaments and scaffolds, were examined using high-performance liquid chromatography and differential scanning calorimetry. All methods have been described in detail previously [82].

The molecular weight and molecular weight distribution of PHA specimens were examined using a size-exclusion chromatograph (1260 Infinity, Agilent Technologies, Waldbronn, Germany) equipped with a DB-35MS column. Molecular weights (weight average, M_w_, and number average, M_n_) and polydispersity (Ð = M_w_/M_n_) were determined.

To determine the thermal properties of PHA specimens, thermal analysis was carried out employing a DSC-1 differential scanning calorimeter (Mettler Toledo, Schwerzenbac, Switzerland) and TGA (Mettler Toledo, Schwerzenbac, Switzerland). The crystallization temperature (T_cryst_) was detected by exothermic peaks, and the glass transition temperature (T_g_), melting point (T_melt_), and thermal degradation temperature (T_degr_) were determined by endothermic peaks on the thermograms. The thermograms were analyzed using the “STARe v11.0” software (Mettler Toledo, Switzerland).

### 3.7. Tensile Testing of Filaments and 3D Scaffolds

The physical and mechanical properties of filaments and scaffolds were studied on an Instron 5565 electromechanical tensile testing machine (Instron, MA, USA). The samples were kept in ambient conditions at 22 °C and 54% humidity for at least two weeks to achieve equilibrium crystallization. Before testing, the dimensions of each sample in different areas were measured using a LEGIONER EDM-25-0.001 electronic digital micrometer (Legioner, Shanghai, China) and taken into account when determining the cross-sectional area. At least five samples were tested for each sample type, and the results were presented as mean ± standard deviation. The measurements were carried out at ambient temperature.

To study the physical and mechanical properties of the filaments, tensile and bending tests were performed. All filament samples were prepared in the form of a cylinder 50 mm long and 1.7 mm in diameter. In the tensile test, the clamp length of the specimens was 30 mm. For the bending test, the type of fastening of specimens was used—a 3-point test. The distance between the supports was 30 mm. The traverse speed in both cases was 3 mm/min.

When studying the physical and mechanical properties of the specimens, compression tests were carried out. In this case, the preload was set equal to 1.0 N. The traverse speed was 1 mm/min. The ultimate strain at the end of the measurement was set to 10%.

The Young’s modulus (E, MPa), ultimate strength (σ, MPa), and elongation at break or flexural strain (ε, %) were calculated using the Bluehill 2 program (Elancourt, France). To obtain the Young’s modulus, the software calculated the slope of each stress–strain curve in its elastic region. The measurement error did not exceed 10%.

### 3.8. In Vitro Assays

The ability of 3D scaffolds to provide and maintain fouling and active cell proliferation was studied in a culture of linear mouse fibroblasts, NIH 3T3. Cultivation was carried out according to the standard method in DMEM medium with the addition of 10% fetal calf serum and 1% antimycotic–antibiotic solution (Gibco, Grand Island, NY, USA) in a humid atmosphere of 5% CO_2_ at 37 °C in a CO_2_ incubator (New Brunswick Scientific, Edison, NJ, USA).

The 3D scaffolds were sterilized using H_2_O_2_ plasma in a Sterrad NX sterilization system (Johnson & Johnson, New Brunswick, NJ, USA). After sterilization, the scaffolds were placed in 24-well culture plates (Corning Costar, New York, NY, USA). NIH 3T3 were seeded at a density of 10^5^ cells per scaffold. The biocompatibility of the studied materials was evaluated in the MTT test based on the reduction of 3-(4,5-dimethylthiazol-2-yl)-2,5-diphenyltetrazolium bromide (Sigma, Pompano Beach, FL, USA) by cellular dehydrogenases to purple MTT formazan crystals. In wells with cells cultured on scaffolds, the medium was changed to a freshly prepared medium containing 5% MTT solution. After 4 h of incubation, the scaffolds were transferred to clean plates, and DMSO was added to dissolve the formed formazan crystals. After 30 min, the stained solutions were transferred to a 96-well plate and the optical density was measured at a wavelength of 550 nm using a Bio-Rad 680 microplate reader (Bio-Rad LABORATORIES Inc., Hercules, CA, USA).

To assess the morphology of NIH 3T3 on scaffolds using electron microscopy, the samples were fixed with 4% glutaraldehyde for 1 h, contrasted with 1% OsO_4_ solution for 1 h, and then dehydrated with a battery of alcohols from 10 to 100%. Before microscopy, the samples were sputtered with platinum and analyzed using a Hitachi TM4000 scanning electron microscope (Hitachi, Tokyo, Japan).

Fluorescent staining of nuclear DNA was performed using the DAPI marker (Sigma-Aldrich, St. Louis, MO, USA) for the visual and quantitative assessment of the formed cell layers. Fluorescent imaging was performed on a Leica DM6000B microscope (Leica, Vienna, Austria) with appropriate LAS software V3.3.

### 3.9. In Vivo Assays

The implantation of 3D scaffolds from P(3HB-co-3HV) was carried out in pigs in accordance with GOST R ISO 10993.6-99, to assess the biological effect of medical devices during implantation, and GOST 53434-2009, which applies to preclinical, non-clinical, and expert studies on the levels of international GLP standards [83,84]. The principles of setting up and performing the experiment were in accordance with ASTM F981-04 (2010) and F1983-99 (2016) for the evaluation of resorbable bone implants [85,86]. The assessment of the biocompatibility of the 3D implants with respect to bone tissue and the level of their osseointegration and biodegradation was carried out using two animals for an implantation period of 5 months [87,88,89].

The experiment was carried out in compliance with the rules of ethics and the humane treatment of animals in the Russian Federation and in the world. The experimental protocol was reviewed and approved by the local ethical committee of the Siberian Federal University, Krasnoyarsk, RF, protocol № 76, 9 September 2021. During implantation, the requirements of the European Convention for the Protection of Vertebrate Animals and Directive 2010/63/EU of the European Parliament were upheld [90,91]. In accordance with the principles of the “3Rs” in Laboratory Animal Science, ‘Replacement, Reduction and Refinement’, of FELASA, the Federation of European Laboratory Animal Science Associations, for the first implantation experiment to test the osteointegration of 3D samples and their biodegradability rates, we took two animals [92]. Male Landrace pigs, 4 months old, purchased from the Shuvaevsky Breeding Plant, a public corporation, with an average weight of 36 ± 1.8 kg, were included in the study. The femur bone was chosen in accordance with the ISO and the goals of the experiment. The animals were maintained in the vivarium at Krasnoyarsk Agricultural University in separate rooms (pens) under standard vivarium conditions of water and diet. The quarantine period was 2 weeks.

Pigs were operated on in the surgery department of the veterinary clinic of Krasnoyarsk Agricultural University. Thirty minutes before surgery, the pigs were anesthetized by an intramuscular injection of Domitor solution 0.1%, as a premedication (0.08 mL/1 kg body mass, Orion Pharma, Espoo, Finland), with atropine sulfate (0.04 mg/1 kg, Moscow Endocrine Plant Federal State Unitary Enterprise, Russia). A combination of Zoletil 100 (6.0 mg/kg, Virbak, Carros, France) and xylazine (2.0 mg/kg, InterchemieWerken “De Adelaar”, Waalre, The Netherlands) was used as a narcoticizing medication. The surgical sites were then shaved and swabbed with 4% chlorhexidine gluconate (Shaanxi Dasheng Chemical Tech., Xi’an, China). The surgery was performed in aseptic conditions and under general anesthesia by an intravenous injection of propofol for narcosis maintenance (1–5 mg/kg/min, Bharat Serums & Vaccines, Maharashtra, India). A longitudinal skin incision was made on the medial side of the right femur. The subcutaneous tissues were moved apart and the periosteum was incised in order to expose the bone surface. One bone cavity was placed in the central femur diaphysis in each animal. These cavities were prepared under sterile saline irrigation (0.9% NaCl) with an orthopedic trephine drill (DIMEDA Instrumente GmbH, Tuttlingen, Germany) at 2000 rpm. Implants were installed in holes, corresponding to the dimensions of the implant’s sizes, manually, with slight effort. After this, the bone wounds were covered with a 2 × 2 cm patch of Bio-Gide resorbable membrane (Geistlich Pharma AG, Wolhusen, Switzerland). Bone wounds were closed with a flap formed from the periosteum and soft tissue and sutured in layers with resorbable interrupted sutures (Vicryl^®^ 0, Ethicon Johnson & Johnson, Somerville, NJ, USA). The skin was sutured with interrupted nonresorbable sutures (ETHILON^®^ 0, Ethicon Johnson & Johnson, Somerville, NJ, USA) and sprayed with aluminum spray (Pharma World 2016 GmbH, Visbek, Germany). For the post-surgery analgesia, Ainil (ketoprofen, 3 mg/1 kg, INVESA, Barselona, Spain, 1 time per day for 3 days) was used. Antimicrobial therapy included Enrofloxacin 5% (2.5 mg/kg, ALPOVET LTD, Limassol, Cyprus) and Bytril 5% (2.5 mg/kg, Bayer Animal Health GmbH, Leverkusen, Germany). Postoperatively, the animals’ condition was monitored, measuring the body temperature, heart rate, and respiration. The sutures were removed after 10 days. Femur bones were resected, and central parts of the diaphysis with implants were sawn out. Circular block sections of the femur were removed and fixed in paraformaldehyde (PFA) for histological analysis. As a control for the bone tissue architecture, we took samples of bone from the diaphysis of the left femur.

### 3.10. Histology

Femur bones were resected, and central parts of the diaphysis with implants were sawn out. Circular block sections of the femur were removed and fixed in paraformaldehyde (PFA) for histological analysis. Bone samples were fixed in 10% neutral formalin one week before decalcification with 0.5 M EDTA in saline (pH 7.4). Sections were taken from the areas of the implants. Samples were then dehydrated using a gradual ethanol series and embedded in paraffin. After cutting the blocks into 6 µm slices, hematoxylin and eosin (H&E) staining was carried out for the subsequent analysis of the bone structure. Microscopy and imaging were performed using AxioScope A1 with AxioCam (Karl Zeiss, Oberkohen, Germany). In the process of microscopic analysis, the condition of the periosteum, cortical layer, and surrounding soft tissue and polymer implant was assessed.

### 3.11. Statistics

Statistical analysis of the results was carried out by conventional methods using the standard Microsoft Excel software package 2019. The presented results were obtained on 3–4 samples, 3 measurements each. High-precision X-ray (3%) and AFM (5%) data were given as averages of 3 measurements of 3 samples. Arithmetic means and standard deviations were given using Student’s *t*-test (significance level: *p* ≤ 0.05).

## 4. Conclusions

The article presents the results of a wide range of studies, including the preparation of a destructible biopolymer from the PHA family (the P(3HB-co-3HV) copolymer), the extrusion production of a filament for 3D printing, the design of 3D scaffolds by FDM technology, and physical–mechanical and biological studies of the obtained polymer products, including testing in cell cultures in vitro and experiments in vivo. Changes in the basic physicochemical properties of the initial P(3HB-co-3HV), obtained granules, filaments, and 3D scaffolds were recorded at all stages of processing, taking into account multi-stage thermal and mechanical effects. The registered changes in the temperature characteristics and molecular weight of the polymer material in the processes of thermal destruction did not adversely affect the process of obtaining and the characteristics of the filaments and scaffolds. The mechanical characteristics of the obtained polymer products were comparable with the literature data. Testing of the 3D scaffolds in an NIH 3T3 fibroblast culture showed no negative effect on cells, which retained their metabolic activity for a long time, actively populated the surface of the scaffold, and penetrated into the structure, filling the spaces between the scaffold elements. In an experiment on large domestic animals (Landrace pigs), the osteoplastic properties of the 3D scaffolds were studied, which were used to fill a model defect in the diaphyseal zone of the femur in the animals. X-ray and histological techniques confirmed the formation of fully mature bone tissue at the site of the model defect and its complete closure within 150 days. The results obtained allow us to conclude that 3D scaffolds constructed from the resorbable P(3HB-co-3HV) copolymer, which do not contain any drugs and osteogenesis stimulators, are suitable for the reconstruction of bone tissue defects and are promising for further research.

## Figures and Tables

**Table 1:** Properties of P(3HB-co-3HV) by stage in the process of processing into filaments and scaffolds.

Sample/Processing Stage	Average Molecular Weight M_w_, кDa	Polydispersity Đ	Melting Temperature T_melt_, °C	Thermal Degradation Temperature T_degr_, °C	Enthalpy of Melting H, J/g	Glass Transition Temperature T_g_, °C	Crystallization Temperature T_cryst_, °C
Original P(3HB-co-3HV)	530	2.3	168	280	98.3	1.1	59/67
Granulate (after the first heating–melting)	480	3.03	149/168/185	279	65.2	1.1	59/62
Filament (after the second heating–melting)	425	2.72	147/168/183	274	73,1	0.3	50/59
3D Scaffold (after the third heating–melting)	390	3.96	161/172/184	275	80,3	0.2	50/54

## Data Availability

The data presented in this study are available on request from the corresponding author. The data are not publicly available due to confidentiality.
